# Multiple systemic embolism in infective endocarditis underlying in Barlow’s disease

**DOI:** 10.1186/s12879-016-1726-5

**Published:** 2016-08-11

**Authors:** Ziqing Yu, Bing Fan, Hongyi Wu, Xiangfei Wang, Chenguang Li, Rende Xu, Yangang Su, Junbo Ge

**Affiliations:** 1 Department of Cardiology, Shanghai Institute of Cardiovascular Diseases, Zhongshan Hospital, Fudan University, 180 Fenglin Road, Shanghai, 200032 People’s Republic of China; 2 Shanghai Medical College, Fudan University, Shanghai, 200032 People’s Republic of China

**Keywords:** Infective endocarditis, Embolism, Antiplatelet treatment

## Abstract

**Background:**

Systemic embolism, especially septic embolism, is a severe complication of infective endocarditis (IE). However, concurrent embolism to the brain, coronary arteries, and spleen is very rare. Because of the risk of hemorrhage or visceral rupture, anticoagulants are recommended only if an indication is present, e.g. prosthetic valve. Antiplatelet therapy in IE is controversial, but theoretically, this therapy has the potential to prevent and treat thrombosis and embolism in IE. Unfortunately, clinical trial results have been inconclusive.

**Case presentation:**

We describe a previously healthy 50-year-old man who presented with dysarthria secondary to bacterial endocarditis with multiple cerebral, coronary, splenic, and peripheral emboli; antibiotic therapy contributed to the multiple emboli. Emergency splenectomy was performed, with subsequent mitral valve repair. Pathological examination confirmed mucoid degeneration and mitral valve prolapse (Barlow’s disease) as the underlying etiology of the endocardial lesion. Continuous antibiotics were prescribed, postoperatively. Transthoracic echocardiography at 1.5, 3, and 6 months after the onset of his illness showed no severe regurgitation, and there was no respiratory distress, fever, or lethargy during follow-up.

**Conclusions:**

Although antibiotic use in IE carries a risk of septic embolism, these drugs have bactericidal and antithrombotic benefits. It is important to consider that negative blood culture and symptom resolution do not confirm complete elimination of bacteria. However, vegetation size and *Staphylococcus aureus* infection accurately predict embolization. It is also important to consider that bacteria can be segregated from the microbicide when embedded in platelets and fibrin. Therefore, antimicrobial therapy with concurrent antiplatelet therapy should be considered carefully.

**Electronic supplementary material:**

The online version of this article (doi:10.1186/s12879-016-1726-5) contains supplementary material, which is available to authorized users.

## Background

Systemic embolism, particularly septic embolism, is a severe complication of IE. However, concurrent embolism to the brain, coronary arteries, and spleen is very rare. Because of the risk of hemorrhage or visceral rupture, anticoagulants are recommended only if an indication for anticoagulation is present [1], e.g. prosthetic valve. Also, antiplatelet therapy in IE remains controversial, with available clinical trials and animal experiments providing contradictory results. Theoretically, antiplatelet therapy has the potential to inhibit and treat thrombosis and embolism in IE. Unfortunately, the results of clinical trials are inconclusive.

## Case presentation

**Fig. 1:**
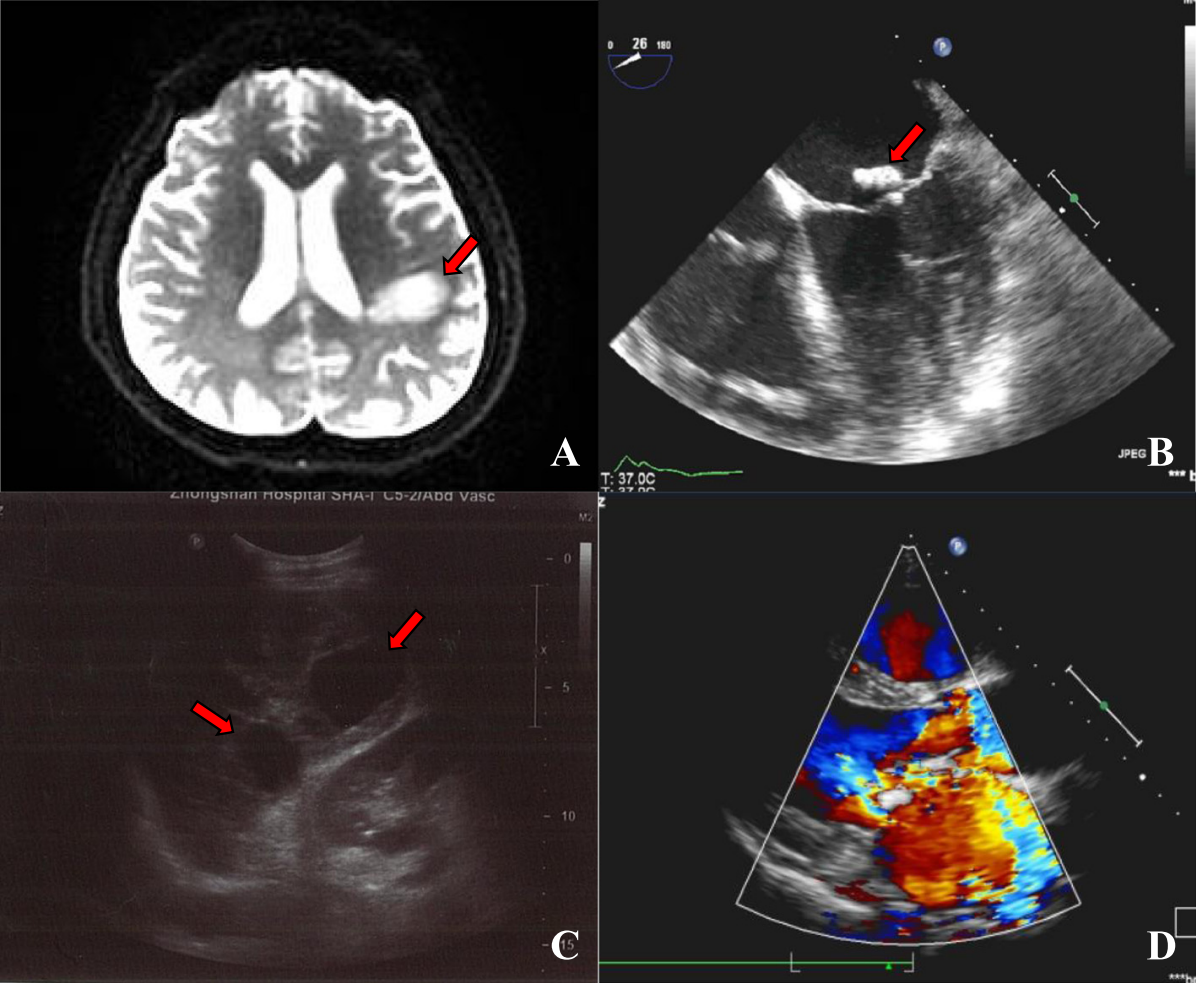
Diagnostic findings. **a**. Cranial T2-magnetic resonance image showing high signal intensity in the left hemisphere (*red arrow*); **b**. Echocardiograph clearly shows a sizeable vegetation on the mitral valve (red arrow); **c**. Abdominal ultrasound showing multiple echo-free areas (*red arrow*) in the spleen, indicating liquefactive necrosis and abscess formation; **d**. Color Doppler showing severe mitral regurgitation

**Fig. 2:**
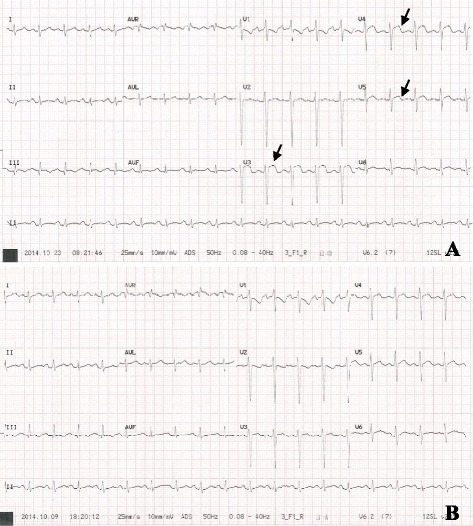
Electrocardiogram tracing. **a**. ST segment elevation in leads V3–V5 (*arrow*) combined with abnormal serum troponin T levels indicated acute coronary syndrome; **b**. ST segment descends gradually to baseline

**Fig. 3:**
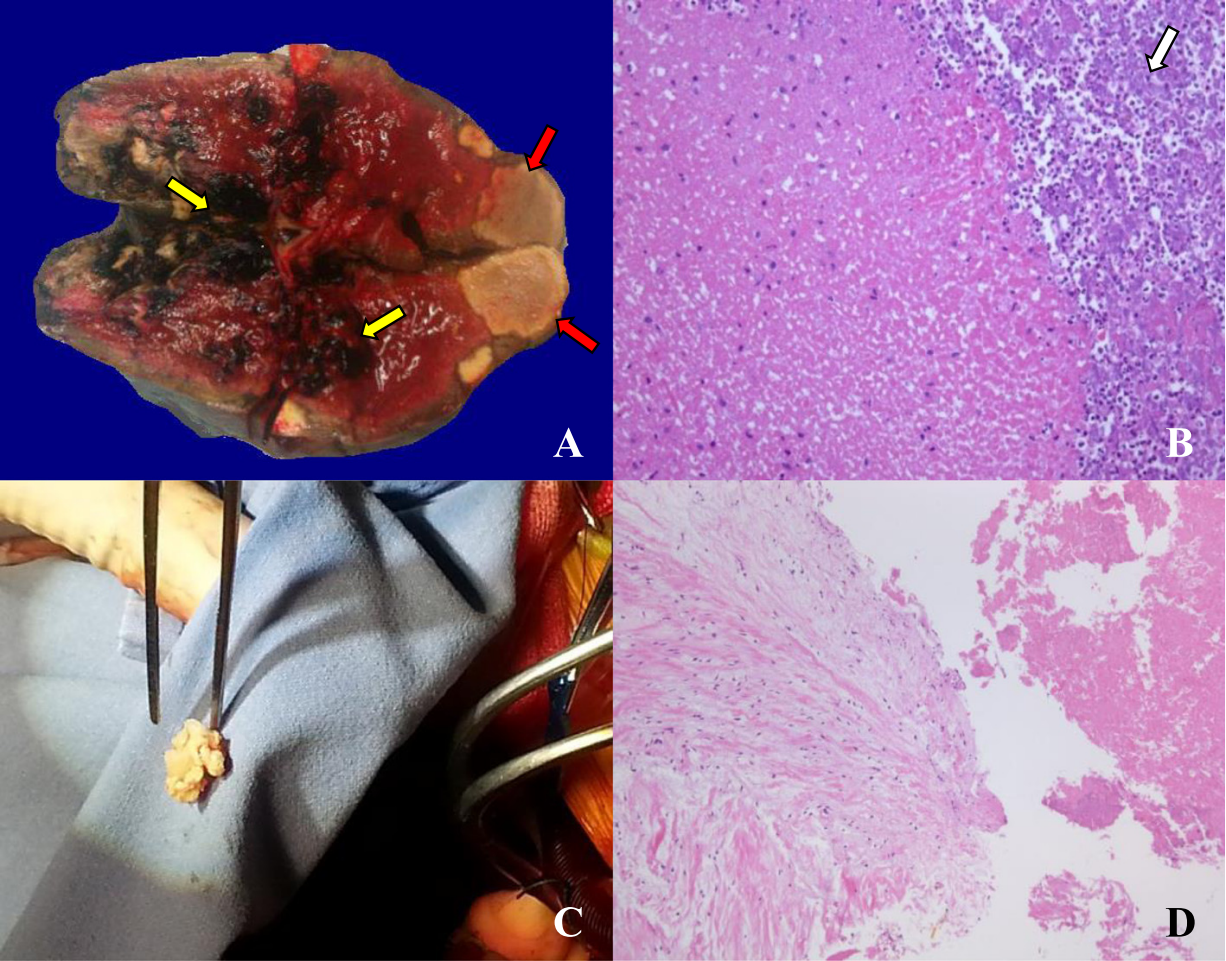
Pathological and gross valve findings. **a**. Necrotic area (*yellow arrow*) and solid abscess (*red arrow*) were seen in a section of removed spleen; b. Necrotic tissue and high neutrophil and macrophage infiltration (*white arrow*) were seen microscopically (magnification, ×200); **c**. Valvular vegetation resected during heart surgery; **d.** Mucoid degeneration of the mitral valve, fibrinous exudation with inflammatory cell infiltration, and mural thrombus formation are seen microscopically (magnification × 100)

A 50-year-old man was admitted to our hospital with acute-onset dysarthria. He presented febrile, with respiratory distress and lethargy, and performed poorly in the right hand alternating movement test. After finding an obvious cardiac murmur, he was transferred to the cardiology department, from the neurology department. Physical examination revealed a blood pressure of 130/90 mm Hg, and heart rate of 116 beats per minute. Cardiac examination revealed that the apex beat was located in the sixth intercostal space, 1.5 cm outside the midclavicular line. Cardiac auscultation revealed a grade 4/6 systolic murmur that was most prominent at the apex. Electrocardiography revealed sinus tachycardia without evidence of ischemia, and serum levels of troponin T, creatine kinase (CK), and CK-myoglobin (CK-MB) were normal; however, N-terminal-pro-brain natriuretic peptide (NT-pro-BNP) levels were increased (3277 pg/ml). The leukocyte count was 10620/mm^3^, with neutrophils at 10 350/mm^3^. The erythrocyte sediment rate was 68 mm/h, and blood culture was positive for group A *β-hemolytic streptococcus*. Transthoracic and transesophageal echocardiography revealed rupture of the mitral chordae tendineae, perforation of the leaflet, mitral vegetation measuring 40 × 30 mm, and severe regurgitation (Fig. 1b and Additional file 1). The left ventricular chamber was enlarged (left ventricular end-diastolic diameter: 64 mm; left ventricular end-systolic diameter: 45 mm), and the pulmonary artery was dilated (diameter: 33 mm; systolic pressure: 94 mm Hg). Cranial MRI revealed high signal intensity in the left hemisphere, indicating cerebral infarction (Fig. 1a). Abdominal ultrasound showed multiple echo-free areas in the spleen that suggested splenic abscess formation (Fig. 1c), and multiple blood cultures confirmed the presence of group A *β-hemolytic Streptococcus*. A diagnosis of left-sided native valve IE was made based on the modified Duke criteria, and the patient’s body temperature was well-controlled by ceftriaxone and vancomycin antibiotic therapy. However, 12 days after presentation, he complained of severe retrosternal chest pain radiating to his back, with ECG showing ST-segment elevation in V3, V4, and V5 leads (Fig. 2a), and increased troponin T (0.542 ng/ml) and CK-MB levels (25 u/l), confirming acute myocardial infarction (AMI). The mechanism of AMI secondary to IE is either compression by aortic periannular lesions or embolism [2]. Because his aorta was not involved, embolism was a concern. Repeat echocardiography showed left ventricular regional and segmental abnormal movement during contraction, supporting AMI. We elected not to initiate antiplatelet therapy, because of his cerebral infarction and splenic abscess, but valve surgery was planned. Unfortunately, 2 days later, he experienced acute-onset sharp abdominal pain with cold limbs, tenderness, muscle tension, guarding, hypotension, and tachycardia, suggesting peritonitis and shock. Bedside ultrasound revealed a 23-mm anechoic area in the abdominal cavity, and intraperitoneal hemorrhage was confirmed by abdominocentesis. Spontaneous splenic rupture was confirmed on emergency laparotomy. A necrotic area was seen in a section of the extracted spleen (Fig. 3a), and necrotic tissue with massive neutrophil and macrophage infiltration was observed microscopically, suggesting abscess formation secondary to septic embolism (Fig. 3b). Mitral valvuloplasty was performed after the patient’s presentation, with vegetation resection, posterior valve repair, and flexible annuloplasty ring placement (Fig. 3c). Pathological analysis revealed mucoid degeneration of the mitral valve, inflammatory cell infiltration, and mural thrombus formation (Fig. 3d). We diagnosed mucoid degeneration and mitral valve prolapse (Barlow’s disease) as the underlying etiology of the endocardial lesion. Transthoracic echocardiography 1.5, 3, and 6 months after the onset of his illness showed no regurgitation, and there was no respiratory distress, fever, or lethargy during follow-up.

## Conclusion

We described here an IE case with multiple systemic embolism in infective endocarditis underlying in Barlow's disease. Although antibiotic use in IE carries a risk of septic embolism, these drugs have bactericidal and antithrombotic benefits. It is important to consider that negative blood culture and symptom resolution do not confirm complete elimination of bacteria. However, vegetation size and Staphylococcus aureus infection accurately predict embolization. It is also important to consider that bacteria can be segregated from the microbicide when embedded in platelets and fibrin. Therefore, antimicrobial therapy with concurrent antiplatelet therapy should be considered carefully.

## Discussion

We report a unique IE case complicated by concurrent cerebral infarction, acute myocardial infarction, and splenic rupture. To our knowledge, a similar case has not been reported previously. In a retrospective study of IE, 499 of 1456 patients (34 %) were complicated by embolic events. Prosthetic valve location, right-sided endocarditis, *Staphylococcus aureus* infection, and vegetation size are considered high risk factors for embolism [3]. In prospective cohorts, embolic events occurred in 34.1 % [4], and 46 % [5] of patients, and age, sex, serum creatinine, and C-reactive protein levels were considered additional risk factors. In other studies [6], the incidence of embolism was 8.5 %, and a formula was developed to determine the likelihood of embolism, considering age, diabetes, atrial fibrillation, embolism before antibiotics, vegetation size, and *Staphylococcus aureus* infection. Results showed that vegetation size and *Staphylococcus aureus* infection validly predict embolization. In septic embolism, antibiotics have dual effects including bactericidal and antithrombotic effects. However, systemic embolism tends to take place in patients with embolism before antibiotic treatment, increasing vegetation size in spite of antimicrobial therapy, when *Staphylococcus spp* are involved in the mitral valve vegetation [7]. Also, antibiotic regimes should be chosen carefully because different antibiotics have different effects on IE. One clinical study revealed that vancomycin and ampicillin were associated with significant reduction in vegetation size; however, cephalosporin and penicillinase-resistant drugs were associated with increased vegetation size and embolic risk [8]. Unfortunately, negative blood culture and resolution of clinical signs do not confirm bacterial elimination. One reason may be drug resistance, and another may be that antibiotics cannot access the center of the vegetation. The vegetation consists of platelets, fibrin, bacteria, and other components, and bacteria are segregated from endogenous microbicides such as some proteins, and exogenous antibiotics, by the platelets and fibrin. The outer crust of the vegetation envelops and confines the bacteria to prevent dissemination, but this protects bacteria from being thoroughly eliminated [9, 10]. It is not currently known which antibiotics perform better regarding anti-thrombosis; therefore, the best future antibiotics will have both good bactericidal effects, and good penetration into the vegetation. Currently, careful consideration should be given to whether concurrent antimicrobial therapy and antiplatelet therapy is more beneficial than antibiotics alone. In vitro, platelet aggregation can be antagonized by aspirin in the presence of bacteria isolated from blood culture [11]. In vivo animal experiments have shown that aspirin has antibacterial effects in IE, inhibiting bacterial adhesion to platelets and vegetation [12]. Aspirin has a dose-dependent effect on reducing vegetation size, with the greatest benefit at middle doses. This is perhaps because lower doses of aspirin do not completely inhibit platelets, while higher doses also decrease prostacyclin, which inhibits platelet aggregation. Regarding antiplatelet effects, aspirin and ticlopidine equally reduce vegetation.

Antiplatelet therapy can dramatically diminish vegetation, when used with vancomycin. However, clinical trials have shown contradictory effects of antiplatelet therapy. Some researchers argue that aspirin reduces stroke events, the number of patients requiring surgery, or mortality, without increasing hemorrhagic events [13–15]; however, these studies were retrospective, and included small sample sizes. A prospective randomized controlled trial showed that aspirin failed to reduce the risk of embolism [16]. Because the cyclooxygenase pathway is not the only way to activate platelets, especially in endocarditis, the activator may not be derived from thromboxane A2; therefore, aspirin, which inhibits cyclooxygenase to reduce thromboxane A2, may play a less important role in IE. This theory may explain why aspirin performed poorly in a prospective trial, and thus, the need for more research regarding other antiplatelet drugs with mechanisms different from aspirin, such as clopidogrel, cilostazol, and ticagrelor, in preventing and treating embolism in IE. Considering the findings in current studies, antiplatelet therapy has potential in IE therapy.

## Abbreviations

AMI, acute myocardial infarction; CK, creatine kinase; IE, infective endocarditis; LVEDD, left ventricular end-diastolic dimension; LVESD, left ventricular end-systolic dimension; NT-pro-BNP, N-Terminal-pro-Brain natriuretic peptide; TXA2, thromboxane A2

## Additional file

Additional file 1: Video Transesophageal echocardiogram during heart surgery (AVI 3770 kb)
